# Use of antipsychotics in adult palliative care: a scoping review protocol

**DOI:** 10.1136/bmjopen-2026-121834

**Published:** 2026-08-24

**Authors:** Anne-Lise Giffard, Emmanuel Mellet, Amandine Mathé

**Affiliations:** 1 Department of Palliative Care, CHU Bordeaux Service d’Accompagnement et de Soins Palliatifs, Bordeaux, France; 2 University of Bordeaux, Talence, France

**Keywords:** PALLIATIVE CARE, Delirium, Adult palliative care

## Abstract

**Abstract:**

**Introduction:**

Antipsychotics are commonly used in adult palliative care practice due to their multiple therapeutic indications. However, the available evidence regarding their use, effectiveness and safety in this context remains heterogeneous, and no comprehensive overview of their use across all palliative indications currently exists. A scoping review is therefore warranted to provide an overall picture of current practices in palliative care. This review aims to synthesise and map the existing evidence regarding the use of antipsychotics in adult palliative care.

**Methods and analysis:**

This scoping review will include studies involving adult human populations (≥18 years) receiving antipsychotics in a palliative care context. All generations of antipsychotics and all palliative indications will be considered. Experimental, observational, qualitative studies, expert recommendations and relevant grey literature published in English or French will be included. The scoping review will be conducted in accordance with the Joanna Briggs Institute methodology and reported in accordance with the Preferred Reporting Items for Systematic Review and Meta-Analysis extension for Scoping Reviews guidelines. Searches will be performed in PubMed, Scopus, PsycInfo, CINAHL, Cochrane Library, Cairn.info and Google Scholar. Two independent reviewers will screen titles, abstracts and full texts and extract data using a standardised form. Results will be synthesised descriptively and presented in tables and narrative form.

**Ethics and dissemination:**

This study does not require ethical approval as it is based on published literature only. The results will be disseminated through publication in a peer-reviewed journal and presentations at scientific conferences.

**STRENGTHS AND LIMITATIONS OF THIS STUDY**

- This scoping review follows Joanna Briggs Institute methodology and will be reported in accordance with the Preferred Reporting Items for Systematic Review and Meta-Analysis extension for Scoping Reviews guidelines, ensuring methodological rigour and transparency.
- A comprehensive search strategy will be conducted across multiple databases and grey literature sources.
- The review will include a broad range of evidence sources, including experimental, observational and qualitative studies, expert recommendations and relevant grey literature.
- Study selection and data extraction will be performed independently by two reviewers using a standardised data charting form.
- No formal risk of bias or quality assessment will be conducted, as the aim is to map the available evidence rather than evaluate study quality.

## Introduction

### Rationale

 Palliative care aims to improve the quality of life of patients and their families facing serious, incurable illness by preventing and relieving physical, psychological, social and spiritual suffering.^1^ As illness progresses, patients frequently experience distressing symptoms requiring complex pharmacological management. Among the medications commonly used in adult palliative care, antipsychotics occupy a particular place because of their wide range of therapeutic indications.

In adult palliative care, antipsychotics are used in several distinct clinical situations, including the management of delirium, nausea and vomiting, pre-existing or newly emerging psychiatric disorders and, in selected situations, palliative sedation.^2–11^ Although these four indications represent the principal therapeutic indications for antipsychotic use in adult palliative care, other less common therapeutic indications have also been described, such as the use of chlorpromazine for the symptomatic management of dyspnoea.^12^

Delirium is one of the most frequent neuropsychiatric syndromes encountered in palliative care patients.^2^ Although non-pharmacological measures remain the cornerstone of management, antipsychotics may be considered when these measures are insufficient, particularly in patients with severe agitation posing a risk to themselves or others, or with distressing perceptual disturbances.^3–5^

Nausea and vomiting are also common symptoms in palliative care.^6^ Several antipsychotics, including haloperidol and levomepromazine (methotrimeprazine), are used for their antiemetic properties in palliative care.^7–9^

Some patients receiving palliative care have pre-existing psychiatric disorders requiring continuation and, when necessary, adaptation of antipsychotic treatment, including changes in formulation or route of administration when the oral route is no longer available, while others may develop psychiatric symptoms during the course of advanced illness.^10^

Antipsychotics may also be used as adjunctive agents in palliative sedation when benzodiazepines alone fail to achieve or maintain adequate sedation.^11^ In France, the Claeys-Leonetti law introduced the right to continuous deep sedation maintained until death for eligible patients experiencing refractory suffering,^13^ while the updated European Association for Palliative Care recommendations also acknowledge the role of antipsychotics in selected situations.^11^

Despite their widespread use across these different clinical contexts, the evidence supporting antipsychotic use in palliative care remains heterogeneous. Several literature reviews have evaluated antipsychotics for individual indications, including nausea and vomiting,^7–9^ delirium^4 5^ and palliative sedation.^11^ However, to our knowledge, no review has comprehensively mapped the use of antipsychotics across all palliative care indications, including psychiatric comorbidities.

Antipsychotics may expose frail palliative care patients to clinically relevant adverse effects, including extrapyramidal symptoms, excessive sedation, QT interval prolongation, orthostatic hypotension, anticholinergic effects and, more rarely, neuroleptic malignant syndrome. In older adults, particularly those living with dementia, antipsychotic use has been associated with an increased risk of mortality,^14^ highlighting the importance of cautious use in frail patients receiving palliative care.

Therefore, a scoping review is warranted to comprehensively map the available evidence, identify knowledge gaps and inform future research regarding the use of antipsychotics in adult palliative care.

### Objectives

This scoping review aims to map and synthesise the available evidence regarding the use of antipsychotics in adult palliative care across all clinical indications, including their indications for use, effectiveness, safety and patterns of use. It also aims to identify gaps in the current literature to inform future research.

The review seeks to answer the following research question: What is the current evidence regarding the use of antipsychotics in adult palliative care?

## Method

This protocol was developed using the Joanna Briggs Institute (JBI) Scoping Review Protocol Template. The scoping review will be conducted in accordance with the methodology described in Chapter 10 of the JBI Manual for Evidence Synthesis.^15^ The completed scoping review will be reported in accordance with the Preferred Reporting Items for Systematic Reviews and Meta-Analyses extension for Scoping Reviews (PRISMA-ScR),^16^ whereas this protocol was prepared using the PRISMA Protocol checklist, adapted for scoping review protocols in accordance with the recommendations of Peters *et al*^17^ (online supplemental appendix 1).

### Inclusion criteria

The inclusion and exclusion criteria for this scoping review were developed following the framework proposed by the JBI, adhering to the Population, Concept and Context (PCC) methodology.^15^ Eligibility criteria are presented within each PCC domain and summarised in the inclusion and exclusion criteria box at the end of this section.

#### Population

This scoping review aims to synthesise evidence related to adult human populations. Studies involving animals or focusing on a paediatric population (under 18 years of age) will be excluded. No eligibility criteria will be applied to participants based on ethnicity, gender identity or sociodemographic characteristics.

#### Concept

Antipsychotics are a heterogeneous class of medications primarily developed for the treatment of psychotic disorders. They are commonly classified into first-generation (typical), second-generation (atypical) and third-generation antipsychotics according to their pharmacological properties. First-generation antipsychotics primarily act through dopamine D2 receptor antagonism, whereas second-generation antipsychotics combine dopamine D2 and serotonin 5-HT2A receptor antagonism. Third-generation antipsychotics, represented by aripiprazole, differ pharmacologically by acting as partial agonists at dopamine D2 and serotonin 5-HT1A receptors and antagonists at serotonin 5-HT2A receptors.^18^

In adult palliative care, antipsychotics are prescribed for a range of clinical indications, including delirium, nausea and vomiting, pre-existing or newly emerging psychiatric disorders, palliative sedation and less commonly, refractory dyspnoea.^2–12^ Their diverse pharmacological profiles contribute to differences in therapeutic effects, adverse effect profiles and prescribing practices across clinical settings.

In the palliative care setting, particular attention should be paid to adverse effects that may have an immediate clinical impact, including extrapyramidal symptoms, QT interval prolongation, drowsiness, excessive sedation, paradoxical agitation, orthostatic hypotension, dizziness, anticholinergic effects, lowering of the seizure threshold and, more rarely, neuroleptic malignant syndrome characterised by muscle rigidity, hyperthermia, altered mental status and autonomic dysfunction.^19–21^

Accordingly, this scoping review will consider all classes of antipsychotics, regardless of generation, molecule, formulation or route of administration, and across all clinical indications relevant to adult palliative care.

No exclusion criteria will be applied regarding the origin of the prescriptions reported in the included studies. No eligibility restrictions will be applied according to the professional background of the prescriber, provided that antipsychotics were prescribed within the legal scope of practice in the country where the study was conducted. Studies assessing the use of antipsychotics in palliative care will be eligible, regardless of the symptoms addressed. No exclusion criteria will be applied based on the targeted indication. Similarly, no exclusion criteria will be applied based on the type of antipsychotic, allowing the inclusion of first-generation, second-generation and third-generation antipsychotics.

#### Context

In this scoping review, the context concerns palliative care.

According to the WHO, palliative care is defined as: ‘Palliative care is an approach that improves the quality of life of patients (adults and children) and their families who are facing problems associated with life-threatening illness. It prevents and relieves suffering through the early identification, correct assessment and treatment of pain and other problems, whether physical, psychosocial or spiritual’.^1^

Nevertheless, supportive care can be defined as ‘the provision of necessary services for those living with or affected by a life-limiting disease to meet their informational, emotional, spiritual, social or physical need during their diagnostic treatment or follow-up phases encompassing issues of health promotion and prevention, survivorship, palliation and bereavement’.^22 23^

Additionally, hospice care is defined as ‘care for the whole person, aiming to meet all needs—physical, emotional, social and spiritual. At home, in day care and in the hospice, they care for the person who is facing the end of life and for those who love them’.^24^ The organisation and delivery of hospice services may vary across healthcare systems, including inpatient hospices, day hospices and community-based services.

Finally, comfort care can be defined as ‘a withdrawal of life-sustaining treatment and/or a plan of care focused primarily on maximizing patient comfort rather than prolonging life’.^25^

For the purpose of this review, a palliative care context will be considered when the study explicitly states that participants received palliative care, hospice care, supportive care with a palliative intent, comfort care or end-of-life care, or when the study was conducted within a specialised palliative care service or programme.

Thus, studies or recommendations published outside the field of palliative care will be excluded. This field may be reflected in journals by terms such as ‘palliative care’, as well as ‘supportive care’, ‘hospice’, ‘hospice care’ and ‘comfort care’. Studies conducted in psychiatry, geriatrics or oncology will be eligible if they explicitly concern patients receiving palliative care. Studies conducted exclusively in these settings without an explicit palliative care context will be excluded.

No exclusion criteria will be applied based on the type of incurable disease affecting patients receiving antipsychotics, including cancer, neurodegenerative diseases or organ failure.

No exclusion criteria will be applied regarding the setting in which antipsychotics are prescribed, provided that the study is conducted in a palliative care context. Studies may therefore be conducted in hospital-based or community-based palliative care settings, with or without collaboration with specialised palliative care teams.

#### Types of documents

In this scoping review, only publications written in French or English will be included, as these are the languages mastered by the investigators, with no restriction on the year of publication.

Both experimental and quasi-experimental studies will be included, encompassing randomised and non-randomised controlled trials, as well as observational studies, such as prospective or retrospective cohort studies, case–control studies and case reports. Qualitative studies will also be included. Literature reviews will be included to map existing evidence. However, if primary studies from these reviews are also included in the final sample, their results will not be extracted to avoid data duplication.

This scoping review will also consider recommendations from expert groups or expert consensus. Articles presenting professional or scientific viewpoints will likewise be considered. Book chapters and documents from the grey literature will be included when deemed relevant to the research question. Conference abstracts will be excluded because they generally provide insufficient methodological and outcome data for reliable data extraction. Theses and dissertations will be considered as grey literature when they meet the eligibility criteria.

**INCLUSION CRITERIA**

- Study involving humans.
- Study involving adults (≥18 years old).
- Study focusing on the use of an antipsychotic, regardless of the generation.
- Study conducted in a palliative care context.

**EXCLUSION CRITERIA**

- Study involving animals.
- Study involving a paediatric population (<18 years old).
- Study not evaluating the use of an antipsychotic.
- Study evaluating the use of an antipsychotic outside the scope of palliative care (eg, general psychiatry, oncology, geriatric medicine).
- Study not available in French or English.
- Study not available in its entirety.

### Source of documents

Various sources and databases were selected based on their relevance to the research question. Electronic databases will be searched from database inception to the date of the final search, without restriction on publication date. First, the following electronic databases will be searched using appropriate search strategies: these include PubMed, SCOPUS, PsycInfo (for the psychosocial field), CINAHL (for the nursing field) and Cairn.info (for the sciences, techniques, medicine, and French-language social sciences and humanities). The Cochrane Library will also be searched.

Additional searches will be conducted using the Google Scholar search module to identify articles from grey literature (the first 100 occurrences).

Finally, the ‘snowball’ method will be employed, which involves manually reviewing the reference lists of the identified articles to find other relevant studies not previously found.

### Search strategy

The search strategy was developed as follows. A first investigator, referred to as A1 in this scoping review, first conducted a preliminary search in PubMed to identify studies on the use of antipsychotics in palliative care and examine the associated keywords. A similar search was conducted on the Google Scholar search engine.

A list of keywords was then created by A1 with the assistance of a second investigator, referred to as A2. Based on these keywords, a search strategy tailored to PubMed was developed and preliminarily validated by A1, A2 and a third investigator (A3), expert in the field of antipsychotics. To assess the reliability and sensitivity of the search strategy, A1 and A2 identified five articles that met the inclusion criteria. The strategy was considered valid if it successfully retrieved all five articles. The search strategy was then definitively validated with the help of a librarian specialising in health sciences.

This search strategy was adapted for use in the other databases employed in this scoping review and validated by A1, A2, A3 and a librarian.

Online supplemental appendix 2 presents the search strategy that will be used for PubMed.

### Selection of articles based on their titles and abstracts

First, the search strategy will be applied to all selected databases (PubMed, SCOPUS, CINAHL, PsycInfo, Cairn.info, Cochrane). The corresponding articles will be identified and collated in the Rayyan software.^26^ The first 100 results of the Google Scholar search will also be integrated into the software. An initial check, with the help of the Rayyan software, will identify and remove duplicates.

A1 and A2 will initiate the selection of articles based on the title and abstract. Before beginning the selection process, a pilot test will be carried out on a randomly selected sample corresponding to 5% of the articles. This will ensure that the two reviewers agree on the use of the selection criteria and definitions associated with key concepts. The pilot test will be considered successful if inter-reviewer agreement is greater than 75%.

Disagreements between A1 and A2 will be resolved through discussion of the eligibility criteria. If the disagreement persists, a third investigator (A3) will be consulted to resolve the conflict.

Based on their titles and abstracts, this selection will be supplemented by articles identified using the snowball method and validated by A1 and A2.

### Selection of articles based on full text

Articles identified based on their title and abstract will then be selected based on their full text by A1 and A2. Similar to the previous step, before starting the selection, a pilot test will be conducted on a randomly selected sample corresponding to 5% of the articles. The pilot test will be considered successful if inter-reviewer agreement is greater than 75%. Disagreements between A1 and A2 will be resolved through discussion of the eligibility criteria. If the disagreement persists, the third investigator (A3) will be consulted to resolve the conflict.

This entire selection process will be summarised in a PRISMA flow chart when this scoping review is published in full.

### Data extraction

For each selected article, the following information will be collected: article title, authors, publication date, journal or website, country, study design, participant characteristics (including age and sex/gender), performance status and estimated life expectancy (when reported), clinical indication for antipsychotic use, including delirium subtype (when applicable), symptom severity and symptom resolution (when reported), type of palliative condition, antipsychotic(s) studied, route of administration, scheduled or as-needed (PRN) prescribing, dose and total daily dose, duration of administration, effectiveness outcomes, safety outcomes, reported adverse effects (including extrapyramidal symptoms, sedation and other adverse events), number of participants and the main study findings. Data will be extracted using a standardised form and presented in summary tables.

Literature reviews and systematic reviews will be included to map the state of knowledge. When primary studies included in these reviews are also included in the final sample, their results will not be extracted to avoid data duplication.

A pilot data extraction test will be carried out by A1 and A2 on approximately 10% of the articles. This will ensure that the information to be collected is consistent. The extraction process will then be carried out independently by the two reviewers. As with the article selection stages, in the event of disagreement, A3 will be asked to make the final decision.

### Data synthesis

As this is a scoping review, no quantitative synthesis will be conducted. Findings will be synthesised using descriptive and narrative approaches, including tabulation of study characteristics, grouping of results into conceptual themes and mapping of the available evidence to identify gaps in the literature.

### Ethics and dissemination

This scoping review will use publicly available data and will not involve the collection of primary data or direct interaction with human participants. As such, ethical approval is not required. The findings will be disseminated through publication in a peer-reviewed scientific journal and presentation at relevant scientific conferences, with the aim of informing clinical practice and identifying gaps in the current evidence on the use of antipsychotics in adult palliative care.

### Patient and public involvement

None.

### Strengths and limitations of this study

This scoping review will use a rigorous methodology, in line with the recommendations of the JBI and the PRISMA-ScR items, ensuring transparency and reproducibility. The search strategy will cover a wide range of sources (multiple databases and grey literature) and include various types of studies (experimental, observational, qualitative), providing a comprehensive overview of practices. The inclusion of all indications for antipsychotics in adult palliative care will also reflect the actual clinical diversity.

However, the scoping review method does not enable a detailed evaluation of the methodological quality of the included studies, which limits the ability to draw conclusions based on high-level evidence. The review will not aim to guide clinical recommendations but only to report descriptively on the different study designs identified.

In addition, language (French and English) and publication biases are possible, and the heterogeneity of contexts, populations and practices may complicate the synthesis of results. Although multiple complementary databases and supplementary search methods will be used, the absence of Embase may have resulted in some relevant studies not being identified. Finally, existing recommendations are often based on expert consensus rather than high-level evidence.

No formal assessment of risk of bias of individual studies will be conducted at either the study or outcome level, as this is a scoping review aiming to map the available evidence rather than assess methodological quality. Similarly, no assessment of meta-biases (eg, publication bias or selective reporting within studies) will be performed. Risk of bias information will not be used in data synthesis. Finally, the certainty of the body of evidence will not be assessed, as the purpose of this scoping review is to comprehensively map the available evidence rather than evaluate its certainty.

## Supplementary material

10.1136/bmjopen-2026-121834 online supplemental appendix 1

10.1136/bmjopen-2026-121834 online supplemental appendix 2

## Data Availability

All data generated and analysed during this study are included in this published article and its supplementary information files, or are available from the corresponding author on reasonable request.
