# Supplementary material for: Use of antipsychotics in adult palliative care: a scoping review protocol
Source: BMJ Open. 2026 Aug 24;16(8):e121834. doi: 10.1136/bmjopen-2026-121834 (PMC13504501; doi:10.1136/bmjopen-2026-121834)
Supplement: Supplementary file 1 [file bmjopen-2026-121834.reviewer_comments.pdf]

PEER REVIEW HISTORY

BMJ Open publishes all reviews undertaken for accepted manuscripts. Reviewers are asked to complete a checklist review form and are provided with free text boxes to elaborate on their assessment. These free text comments are reproduced below.

ARTICLE DETAILS

Title (Provisional)

Use of antipsychotics in adult palliative care: a scoping review protocol

Authors

GIFFARD, Anne-Lise; Mellet, Emmanuel; Mathé, Amandine

VERSION 1 - REVIEW

**Reviewer**	**1**
**Name**	**Bush, Shirley H.**
**Affiliation**	**University of Ottawa, Department of Medicine**
**Date**	**12-Jun-2026**
**COI**	**None**

I have the following comments:

1. The Title uses the term “Antipsychotic prescription”, but an antipsychotic (AP) could be prescribed but end up not being administered/ used by a patient. As you are not studying the actual prescription of APs, perhaps the title should be changed to something like “antipsychotic utilisation” or “use of antipsychotics” (as used in Abstract objective) or “receipt of antipsychotics” (as used in Abstract Inclusion criteria) to better describe the focus of this Scoping Review.

2. In Abstract Introduction (Line 40), the word “efficacy” is used. “Effectiveness” may be a better term as effectiveness reflects an intervention’s ‘real world’ performance. This contrasts with efficacy studies which occur in a highly selected sub-population who are unlikely to have other comorbidities or take other medications.

3. In Abstract Methods (line 55), it is stated that MEDLINE will be searched. However, in the manuscript, you write that you are searching PubMed, as opposed to ‘MEDLINE via Ovid’ (which is often used by health science librarians and researchers for comprehensive systematic searches). So for clarity, suggest changing in both Abstract and protocol Methods (e.g. Page 8, Lines 31 + 45 + 51, Page 9, Line 7 + 12), and also in Appendix 2 title, that PubMed will be searched (without including MEDLINE wording).

4. Looking at the list of databases to be searched as part of this scoping review, I was surprised that the Embase database was not included given the topic of the review on antipsychotics, considering that Embase is a biomedical and pharmacological database. What is your rationale for not including Embase?

5. It is good to see that the authors plan to report using PRISMA-ScR guidelines.

The protocol flow could be improved if the same subheadings were used in this protocol as used in PRISMA-ScR: https://www.prisma-statement.org/scoping

The Introduction section would then have the subheadings of Rationale and Objectives and contain the checklist-described content in those sections. The current Introduction section currently focuses more on the Concept.

The "Best practice guidance and reporting items for the development of scoping review protocols" published in 2022 by Peters and other members of the JBI Scoping Review Methodology Group provides 'Recommended items to report in a scoping review protocol' mapped against PRISMA-P as Table 1: https://pubmed.ncbi.nlm.nih.gov/35102103/

It will be useful for the authors to use this resource to finalise their completed PRISMA-P checklist (Appendix 1 and separate attachment).

In the absence of a PRISMA-ScR protocol checklist at this time, I looked up the JBI template for Scoping Review protocols. (It is available here: https://jbi.global/scoping-review-network/resources). It looks as if the authors may have used this as a template to write this ScR protocol. If this is the case, then the authors should state this.

6. Throughout the protocol, the terminology used alters between "palliative care" and "palliative medicine."

Suggest using one term ("palliative care") throughout.

On reading this ScR protocol, I was not always clear what steps had already been completed e.g. "electronic databases will be searched" versus "Cochrane database was also searched" in the same paragraph on Page 8. Suggest checking the tense of verbs that are used to make it clear what steps of the protocol have already been completed (in past tense) and what steps are still to be completed using future tense.

7. Page 4, line 3: The authors write that there are four major groups of indications for the prescription of antipsychotics. In Abstract, Line 41: the authors write that there is "no comprehensive overview of their (i.e. AP) use across all palliative indications." Another less common role for chlorpromazine is for the symptom management of dyspnoea, and by extension also for levomepromazine (also known as methotrimeprazine) as this is also a phenothiazine. Link for 1994 article in JPSM: https://pubmed.ncbi.nlm.nih.gov/7963786/

8. Delirium is not simply 'mental confusion' so this description should be removed.

Page 4, Line 8: Suggest: "Delirium is a common symptom in adult palliative care patients."

Page 5, Lines 3-4: Suggest: “Similarly, a 2018 Cochrane review examined the use of antipsychotics for delirium in patients hospitalised outside intensive care units.”

9. Page 4, Lines 14-16.

Hallucinations and delusions are not specific to the hyperactive subtype of delirium.

Hallucinations, illusions, and delusions can occur in both hyperactive and hypoactive subtypes, but they are more common in hyperactive delirium.

The authors do not mention mixed delirium which has fluctuating features of both hyperactive and hypoactive delirium.

As per delirium guideline recommendations, antipsychotics (on a short-term basis) may have a role in the management of distressing perceptual disturbances (for all delirium subtypes) that cannot be managed by non-pharmacological interventions, or if the patient is severely agitated and a potential risk to themselves or to others.

10. Page 4, lines 21-25.

While metoclopramide is a dopamine D2 receptor antagonist (as are many antipsychotics), it does not “belong to the antipsychotic class of drugs” as it is not an antipsychotic.

Levomepromazine is also known as methotrimeprazine in some countries so this alternative name should be added.

The antiemetic prochlorperazine could also be considered – it is a phenothiazine and thus in the same class as levomepromazine (methotrimeprazine) but much less sedating.

11. Page 4, Lines 29-30:

“For these patients, treatment with antipsychotics must continue”. As the originally prescribed AP may be an oral formulation, it will be important to capture if it is changed to an AP that is available as a parenteral formulation, given the loss of oral route at the end of life.

12. Page 5: lines 18-19 and 22:

It is challenging to categorically state that there is no previous literature review or no previous study, as a review or study may in fact exist, but the authors did not find it on a literature review.

As such, suggest adding “to our knowledge” for both these lines.

13. Page 5: lines 24-26

The authors could highlight the risk of harm in using antipsychotics in older people, and the increased risk of death in people with dementia.

14. Page 6: Concept paragraph.

For lines 13-14: While the first-generation antipsychotics are dopamine D2 antagonists, the second-generation antipsychotics are 5-HT2A and dopamine D2 antagonists and so do not primarily target the dopaminergic system. Aripiprazole (a third-generation antipsychotic) does not block central dopaminergic receptors as it is a dopamine D2 partial agonist, 5-HT1A partial agonist, and 5-HT2A antagonist. (Lines 30-32: third-generation antipsychotics not included as part of classification).

The Concept paragraph focuses on the role of antipsychotics for people with schizophrenia, as opposed to giving a description of their role in palliative care patients (for delirium, palliative sedation, nausea + vomiting, dyspnoea). Perhaps some of the Introduction paragraph should be moved to the Concept paragraph to make the protocol feel less disjointed?

The Concept paragraph describes in detail the side effects of antipsychotics when used long term (such as in people with schizophrenia), e.g. metabolic and endocrine effects which would not usually be relevant for palliative care patients where APs are used for a shorter period of time.

It would be of value to focus the Concept paragraph on potential adverse effects of APs that would be of concern for palliative care patients. e.g. EPS, QTc interval prolongation, drowsiness, sedation, paradoxical agitation, orthostatic hypotension, dizziness, anticholinergic effects, neuroleptic malignant syndrome, lowering of seizure threshold (especially with sedating first-generation APs), anxiety, insomnia.

For neuroleptic malignant syndrome, most authors describe it as “muscle rigidity” as opposed to “extrapyramidal rigidity”.

It would be helpful to include citations for the Concept paragraph.

15. Page 6: lines 35-37. This would fit better under the heading “Aims of review” on Page 5. Also important to include assessment of evidence regarding safety of APs for adult palliative care patients.

16. Page 6: lines 40-47. These lines describe Exclusion criteria so should be part of Inclusion/ Exclusion criteria paragraph on Page 5. In addition to “licensed physician” prescribing, would nurse practitioners prescribe APs?

Also, the Box detailing Inclusion/ Exclusion criteria should be moved to the relevant section on Page 5. Age in Box should be corrected to “≥ 18 years old” (as in Abstract).

For “Palliative Care context”, how will this be determined?

For listed exclusion of “oncology”, does this mean that patients at the end of life looked after on the oncology ward will be excluded?

Are you including or excluding conference abstracts, books, theses?

17: Page 7: lines 10-12. Hospice care needs to be redefined. The current description for hospice care is only relevant to US populations (the cited paper is from the US). In the US,

hospice care is predominantly community based due to the US Hospice Benefit. However, for other countries, “hospices” are standalone inpatient facilities. As such, the term “hospice” should be included, not just “hospice care” in Line 20 (Page 7).

Page 7: Lines 17-33 describe exclusion criteria and should be part of Inclusion/Exclusion criteria section.

Page 8: Method, Lines 19-24: This section needs correction. I was not able to find a “PRISMA-ScR protocol”, only the PRISMA-ScR Checklist that is to be used in writing up the completed ScR. Appendix 1 is for the PRISMA-P checklist for systematic review protocols, and not ScR protocols.

For the online version of the JBI Manual for Evidence Synthesis, Scoping Reviews are Chapter 10, and the section was last updated in March 2024: https://jbi-global-wiki.refined.site/space/MANUAL/355862497/10.+Scoping+reviews

As such, it would seem prudent to use this more up-to-date online resource than citation 17 in conducting this scoping review.

Source of documents: The dates for the planned literature search should be included. E.g. from inception for the database searches?

Lines 56-57: Are the authors planning to do a peer review of the search strategy (PRESS) by another academic librarian?

18. Data extraction, Page 9. This should align with Page 5, lines 24-26. So anticipated side effects (including EPS and sedation) should be listed here, as well as Safety.

Participant age and sex/gender should be included for data extraction.

It would also be interesting to see if patients’ performance status or estimated life expectancy are reported.

Other data to consider: delirium subtype, symptom severity, and symptom resolution.

In addition to AP doses, it would be useful to know if the AP was PRN (as needed) or scheduled dosing, and total daily dose.

Some additional minor comments:

1. Suggest adding “Scoping review” as a key word. (“Scoping review” is a MeSH term in PubMed).

2. In abstract and throughout the manuscript, the wording “Joanna Briggs Institute” is used. The organization changed its name to simply “JBI”, so “Joanna Briggs Institute” should be removed throughout. (https://jbi.global/)

3. Page 3, line 13: Strengths and limitations section: the word “international” to describe the databases is not needed. You can simply state “multiple databases”.

4. Page 4, Lines 38-41: Please add citation for the 2016 law.

5. Page 5, Line 5: It is not usual to include the first name of an author in academic writing.

6. Page 9, Line 15: the citation for Rayyan software should be given. Here is the link I found on the Rayyan website which may be helpful: https://help.rayyan.ai/hc/en-us/articles/45541638273681-Citing-Rayyan-in-Your-Research

7. Page 9: Lines 25 and 43 and Page 10: line 11: For "Peters et al, (2020)", citation number should be added (is it citation #12?)

VERSION 1 - AUTHOR RESPONSE

- **Reviewer – Comment 1**

The Title uses the term "Antipsychotic prescription", but an antipsychotic (AP) could be prescribed but end up not being administered/ used by a patient. As you are not studying the actual prescription of APs, perhaps the title should be changed to something like "antipsychotic utilisation" or "use of antipsychotics" (as used in Abstract objective) or "receipt of antipsychotics" (as used in Abstract Inclusion criteria) to better describe the focus of this Scoping Review.

Response 1

Thank you for this helpful comment. We agree that the term *prescription* may be too restrictive, as this scoping review aims to map the broader use of antipsychotics in adult palliative care rather than prescribing practices alone. Accordingly, the title has been revised to: "Use of antipsychotics in adult palliative care: a scoping review protocol." The terminology has also been harmonised throughout the manuscript where appropriate.

- **Reviewer – Comment 2**

In Abstract Introduction (Line 40), the word "efficacy" is used. "Effectiveness" may be a better term as effectiveness reflects an intervention's 'real world' performance. This contrasts with efficacy studies which occur in a highly selected sub-population who are unlikely to have other comorbidities or take other medications.

Response 2

We agree with the reviewer that *effectiveness* is a more appropriate term than *efficacy* in the context of this scoping review, which aims to map evidence from a broad range of study designs reflecting real-world clinical practice. The abstract has been revised accordingly.

- **Reviewer – Comment 3**

In Abstract Methods (line 55), it is stated that MEDLINE will be searched. However, in the manuscript, you write that you are searching PubMed, as opposed to 'MEDLINE via Ovid' (which is often used by health science librarians and researchers for comprehensive systematic

searches). So for clarity, suggest changing in both Abstract and protocol Methods (e.g. Page 8, Lines 31 + 45 + 51, Page 9, Line 7 + 12), and also in Appendix 2 title, that PubMed will be searched (without including MEDLINE wording).

Response 3

We agree with the reviewer that this terminology should be clarified. As the search strategy was developed and will be conducted using PubMed, rather than MEDLINE via Ovid, the manuscript has been revised accordingly. The term PubMed has been used consistently throughout the manuscript and in Appendix 2.

- **Reviewer – Comment 4**

Looking at the list of databases to be searched as part of this scoping review, I was surprised that the Embase database was not included given the topic of the review on antipsychotics, considering that Embase is a biomedical and pharmacological database. What is your rationale for not including Embase?

Response 4

We thank the reviewer for this important comment. We agree that Embase is a major biomedical and pharmacological database and that its inclusion would have strengthened the comprehensiveness of the search strategy. Unfortunately, Embase was not available through our institutions despite verification with our university library services. To maximise the comprehensiveness of the review, we included several complementary databases (PubMed, Scopus, PsycINFO, CINAHL, Cochrane Library, and Cairn.info), supplemented by Google Scholar and backward citation searching. We have also acknowledged the absence of Embase as a limitation of the review in the Strengths and limitations section.

- **Reviewer – Comment 5**

It is good to see that the authors plan to report using PRISMA-ScR guidelines. The protocol flow could be improved if the same subheadings were used in this protocol as used in PRISMA-ScR:https://www.prisma-statement.org/scoping The Introduction section would then have the subheadings of Rationale and Objectives and contain the checklist-described content in those sections. The current Introduction section currently focuses more on the Concept.

The "Best practice guidance and reporting items for the development of scoping review protocols" published in 2022 by Peters and other members of the JBI Scoping Review Methodology Group provides 'Recommended items to report in a scoping review protocol' mapped against PRISMA-P as Table 1: https://pubmed.ncbi.nlm.nih.gov/35102103/ It will be useful for the authors to use this resource to finalise their completed PRISMA-P checklist (Appendix 1 and separate attachment).

In the absence of a PRISMA-ScR protocol checklist at this time, I looked up the JBI template for Scoping Review protocols. (It is available here: https://jbi.global/scoping-review-

network/resources). It looks as if the authors may have used this as a template to write this ScR protocol. If this is the case, then the authors should state this.

Response 5

We appreciate the reviewer's thoughtful suggestions. The manuscript has been revised to improve its alignment with current guidance for scoping review protocols. The Introduction has been restructured using the subheadings Rationale and Objectives, and the objectives have been streamlined to reduce redundancy. We have also clarified the methodological framework by explicitly stating that the protocol was informed by the JBI Scoping Review Protocol Template, will be conducted in accordance with the JBI Manual for Evidence Synthesis, and that the completed scoping review will be reported following the PRISMA-ScR guidelines. In addition, the completed PRISMA-P checklist has been revised, where appropriate, in accordance with the recommendations of Peters et al. (2022).

- **Reviewer – Comment 6**

Throughout the protocol, the terminology used alters between "palliative care" and "palliative medicine." Suggest using one term ("palliative care") throughout.

On reading this ScR protocol, I was not always clear what steps had already been completed e.g. "electronic databases will be searched" versus "Cochrane database was also searched" in the same paragraph on Page 8. Suggest checking the tense of verbs that are used to make it clear what steps of the protocol have already been completed (in past tense) and what steps are still to be completed using future tense.

Response 6

We thank the reviewer for this helpful suggestion. The terminology has been standardised throughout the manuscript by consistently using the term "palliative care". In addition, verb tenses have been harmonised to clearly distinguish procedures that have already been completed (e.g. development and validation of the search strategy) from those that will be undertaken during the conduct of the scoping review.

- **Reviewer – Comment 7**

Page 4, line 3: The authors write that there are four major groups of indications for the prescription of antipsychotics. In Abstract, Line 41, the authors write that there is "no comprehensive overview of their (i.e. AP) use across all palliative indications." Another less common role for chlorpromazine is for the symptom management of dyspnea, and by extension also for levomepromazine (also known as methotrimeprazine) as this is also a phenothiazine. Link for 1994 article in JPSM: https://pubmed.ncbi.nlm.nih.gov/7963786/

Response 7

We thank the reviewer for highlighting this additional indication. We agree that, although delirium, nausea and vomiting, psychiatric disorders, and palliative sedation represent the principal therapeutic indications for antipsychotic use in adult palliative care, other less common indications have also been reported. We have therefore revised both the Introduction and the Concept section to acknowledge the use of chlorpromazine for the symptomatic

management of refractory dyspnoea and have added the suggested reference (Bruera et al., 1994).

- **Reviewer – Comment 8**

Delirium is not simply 'mental confusion' so this description should be removed. Page 4, Line 8: Suggest: "Delirium is a common symptom in adult palliative care patients." Page 5, Lines 3-4: Suggest: "Similarly, a 2018 Cochrane review examined the use of antipsychotics for delirium in patients hospitalised outside intensive care units."

Response 8

We thank the reviewer for this helpful suggestion. The expression "mental confusion" has been removed and replaced by the term "delirium" throughout the manuscript. The Introduction has also been revised to describe delirium as a common symptom in adult palliative care patients. In addition, the description of the 2018 Cochrane review has been updated to refer consistently to delirium, in accordance with the reviewer's suggestion.

- **Reviewer – Comment 9**

Page 4, Lines 14-16. Hallucinations and delusions are not specific to the hyperactive subtype of delirium. Hallucinations, illusions, and delusions can occur in both hyperactive and hypoactive subtypes, but they are more common in hyperactive delirium. The authors do not mention mixed delirium which has fluctuating features of both hyperactive and hypoactive delirium. As per delirium guideline recommendations, antipsychotics (on a short-term basis) may have a role in the management of distressing perceptual disturbances (for all delirium subtypes) that cannot be managed by non-pharmacological interventions, or if the patient is severely agitated and a potential risk to themselves or to others.

Response 9

We thank the reviewer for this important clarification. The Introduction has been revised to remove the previous description linking specific symptoms to delirium motor subtypes. It now reflects current guideline recommendations by emphasizing that non-pharmacological measures remain the cornerstone of delirium management and that antipsychotics may be considered when these measures are insufficient, particularly in patients with severe agitation posing a risk to themselves or others, or with distressing perceptual disturbances.

- **Reviewer – Comment 10**

Page 4, lines 21-25. While metoclopramide is a dopamine D2 receptor antagonist (as are many antipsychotics), it does not "belong to the antipsychotic class of drugs" as it is not an antipsychotic. Levomepromazine is also known as methotrimeprazine in some countries so this alternative name should be added. The antiemetic prochlorperazine could also be considered – it is a phenothiazine and thus in the same class as levomepromazine (methotrimeprazine) but much less sedating.

Response 10

We thank the reviewer for these helpful suggestions. The Introduction has been revised to remove the inaccurate statement that metoclopramide belongs to the antipsychotic class of drugs. We have also added the alternative name methotrimeprazine for levomepromazine. We

appreciate the suggestion to include prochlorperazine. However, to keep the Introduction concise and focused on representative examples of antipsychotics used in palliative care, we chose not to discuss additional individual agents.

- **Reviewer – Comment 11**

Page 4, Lines 29-30: "For these patients, treatment with antipsychotics must continue". As the originally prescribed AP may be an oral formulation, it will be important to capture if it is changed to an AP that is available as a parenteral formulation, given the loss of oral route at the end of life.

Response 11

We thank the reviewer for this valuable suggestion. The Introduction has been revised to acknowledge that antipsychotic treatment may require adaptation, including changes in formulation and/or route of administration when the oral route is no longer available. In addition, information on the route of administration will be collected during data extraction, where reported.

- **Reviewer – Comment 12**

Page 5: lines 18-19 and 22: *It is challenging to categorically state that there is no previous literature review or no previous study, as a review or study may in fact exist, but the authors did not find it on a literature review. As such, suggest adding "to our knowledge" for both these lines.*

Response 12

We thank the reviewer for this helpful suggestion. We agree that categorical statements should be avoided. The relevant statements have been revised accordingly, including the addition of "to our knowledge" where appropriate.

- **Reviewer – Comment 13**

Page 5: lines 24-26. The authors could highlight the risk of harm in using antipsychotics in older people, and the increased risk of death in people with dementia.

Response 13

We thank the reviewer for this valuable suggestion. The Introduction has been revised to highlight the potential risks associated with antipsychotic use in frail palliative care patients. We now also note that, in older adults, particularly those living with dementia, antipsychotic use has been associated with an increased risk of mortality, supporting the need for cautious prescribing in frail palliative care populations. An additional reference has been added to support this statement.

- **Reviewer – Comment 14**

Page 6: Concept paragraph.

For lines 13-14: While the first-generation antipsychotics are dopamine D2 antagonists, the second-generation antipsychotics are 5-HT2A and dopamine D2 antagonists and so do not primarily target the dopaminergic system. Aripiprazole (a third-generation antipsychotic) does

not block central dopaminergic receptors as it is a dopamine D2 partial agonist, 5-HT1A partial agonist, and 5-HT2A antagonist. (Lines 30-32: third-generation antipsychotics not included as part of classification).

The Concept paragraph focuses on the role of antipsychotics for people with schizophrenia, as opposed to giving a description of their role in palliative care patients (for delirium, palliative sedation, nausea + vomiting, dyspnoea). Perhaps some of the Introduction paragraph should be moved to the Concept paragraph to make the protocol feel less disjointed? The Concept paragraph describes in detail the side effects of antipsychotics when used long term (such as in people with schizophrenia), e.g. metabolic and endocrine effects which would not usually be relevant for palliative care patients where APs are used for a shorter period of time. It would be of value to focus the Concept paragraph on potential adverse effects of APs that would be of concern for palliative care patients, e.g. EPS, QTc interval prolongation, drowsiness, sedation, paradoxical agitation, orthostatic hypotension, dizziness, anticholinergic effects, neuroleptic malignant syndrome, lowering of seizure threshold (especially with sedating first-generation APs), anxiety, insomnia.

For neuroleptic malignant syndrome, most authors describe it as "muscle rigidity" as opposed to "extrapyramidal rigidity".

It would be helpful to include citations for the Concept paragraph.

<u>Response 14</u>

We thank the reviewer for these thoughtful and constructive comments. In response, we have substantially revised the Concept section to better reflect the scope of this scoping review and current pharmacological knowledge. The section now provides a concise description of first-, second-, and third-generation antipsychotics, including the distinct pharmacological profile of aripiprazole. It has been refocused on the role of antipsychotics in adult palliative care by describing the principal clinical indications relevant to this review. We have also revised the description of adverse effects to emphasise those most relevant in palliative care, including extrapyramidal symptoms, QT interval prolongation, sedation, orthostatic hypotension, anticholinergic effects, lowering of the seizure threshold and neuroleptic malignant syndrome. Finally, appropriate supporting references have been added throughout the section.

- **Reviewer – Comment 15**

Page 6: lines 35-37. This would fit better under the heading "Aims of review" on Page 5. Also important to include assessment of evidence regarding safety of APs for adult palliative care patients.

<u>Response 15</u>

We thank the reviewer for this helpful suggestion. We agree that the statement describing the purpose of the review was more appropriately placed under the Objectives section than within the Concept section. It has therefore been relocated and the objectives have been revised accordingly. In addition, the objectives now explicitly include the assessment of the available evidence regarding the safety of antipsychotics in adult palliative care.

- **Reviewer – Comment 16**

These lines describe Exclusion criteria and should be part of the Inclusion/Exclusion criteria paragraph on Page 5. In addition to "licensed physician" prescribing, would nurse practitioners prescribe APs? Also, the Box detailing Inclusion/Exclusion criteria should be moved to the relevant section on Page 5. Age in Box should be corrected to "≥ 18 years old" (as in Abstract). For "Palliative Care context", how will this be determined? For listed exclusion of "oncology", does this mean that patients at the end of life looked after on the oncology ward will be excluded? Are you including or excluding conference abstracts, books, theses?

Response 16

We thank the reviewer for this helpful comment. To improve clarity, we have clarified that eligibility criteria are presented within each domain of the JBI Population–Concept–Context (PCC) framework and are subsequently summarised in the inclusion and exclusion criteria box. This reflects the structure recommended by the PCC framework while providing a concise overview of the eligibility criteria.

We agree that prescribing authority varies across healthcare systems. The manuscript has therefore been revised to clarify that no eligibility restrictions apply according to the professional background of the prescriber, provided that antipsychotics were prescribed within the legal scope of practice in the country where the study was conducted.

The inclusion and exclusion criteria summary box is presented immediately after the eligibility criteria sections (Population, Concept, Context and Types of documents). To improve clarity, we have further clarified in the introductory paragraph that eligibility criteria are described within each PCC domain and summarised in the inclusion and exclusion criteria box.

We thank the reviewer for identifying this inconsistency. The age criterion in the inclusion criteria box has been corrected from >18 years to ≥18 years, in line with the abstract and the Population section.

The manuscript has been revised to clarify how a palliative care context will be determined during study selection. We now specify that studies will be considered eligible when they explicitly describe palliative care, hospice care, supportive care with a palliative intent, comfort care, end-of-life care, or when they are conducted within a specialised palliative care service or programme.

The manuscript has been revised to clarify that studies conducted in oncology, psychiatry or geriatrics are eligible when they explicitly concern patients receiving palliative care. Only studies conducted outside a palliative care context will be excluded.

The eligibility criteria for document types have been clarified. We now explicitly state that conference abstracts will be excluded because they generally provide insufficient information for reliable data extraction. We also clarify the eligibility of book chapters and grey literature.

- **Reviewer – Comment 17**

Page 7: lines 10–12. Hospice care needs to be redefined. The current description for hospice care is only relevant to US populations (the cited paper is from the US). In the US, hospice care is predominantly community based due to the US Hospice Benefit. However, for other

countries, "hospices" are standalone inpatient facilities. As such, the term "hospice" should be included, not just "hospice care" in Line 20 (Page 7).

Page 7: Lines 17–33 describe exclusion criteria and should be part of Inclusion/Exclusion criteria section.

Page 8: Method, Lines 19–24: This section needs correction. I was not able to find a "PRISMA-ScR protocol", only the PRISMA-ScR Checklist that is to be used in writing up the completed ScR. Appendix 1 is for the PRISMA-P checklist for systematic review protocols, and not ScR protocols. For the online version of the JBI Manual for Evidence Synthesis, Scoping Reviews are Chapter 10, and the section was last updated in March 2024: https://jbi-global-wiki.refined.site/space/MANUAL/355862497/10.+Scoping+reviews As such, it would seem prudent to use this more up-to-date online resource than citation 17 in conducting this scoping review.

Source of documents: The dates for the planned literature search should be included. E.g. from inception for the database searches?

Lines 56–57: Are the authors planning to do a peer review of the search strategy (PRESS) by another academic librarian?

Response 17

We thank the reviewer for this important observation. The definition of hospice has been revised to avoid a US-specific perspective and to better reflect international models of hospice services. We have also explicitly included both the terms *"hospice"* and *"hospice care"* when defining the palliative care context.

As clarified above (Comment 16), eligibility criteria are presented within each domain of the JBI Population–Concept–Context (PCC) framework and are summarised in the inclusion and exclusion criteria box.

The *Methods* section has been revised accordingly to reflect the current JBI guidance for scoping reviews. We now explicitly state that this protocol was developed using the JBI Scoping Review Protocol Template and will be conducted in accordance with the current JBI methodology for scoping reviews (Chapter 10 of the JBI Manual for Evidence Synthesis). We also clarify that the completed scoping review will be reported according to the PRISMA-ScR guidelines, whereas this protocol was prepared using the PRISMA-P checklist adapted for scoping review protocols, in accordance with the recommendations of Peters et al.

The *Source of Documents* section has been revised to specify the planned search period. We now state that all electronic databases will be searched from database inception to the date of the final search, without restriction on publication date.

We thank the reviewer for this suggestion. The search strategy was developed and validated with the support of a health sciences librarian. However, a formal PRESS (Peer Review of Electronic Search Strategies) review is not planned for this scoping review.

- **Reviewer – Comment 18**

Data extraction, Page 9. This should align with Page 5, lines 24–26. So anticipated side effects (including EPS and sedation) should be listed here, as well as Safety. Participant age and sex/gender should be included for data extraction. It would also be interesting to see if patients' performance status or estimated life expectancy are reported. Other data to consider: delirium subtype, symptom severity, and symptom resolution. In addition to AP doses, it would be useful to know if the AP was PRN (as needed) or scheduled dosing, and total daily dose.

Response 18

The *Data extraction* section has been revised to better align with the objectives of this scoping review and with the variables described in the *Concept* section. The data extraction form has been expanded to include participant characteristics (age and sex/gender), performance status and estimated life expectancy (when reported), clinical characteristics (including delirium subtype, symptom severity and symptom resolution, when applicable), detailed antipsychotic prescribing information (scheduled or PRN administration, dose and total daily dose), as well as effectiveness outcomes, safety outcomes and reported adverse effects, including extrapyramidal symptoms and sedation, when available.

- **Minor comment 1**

The keyword "Scoping review" has been added to improve indexing and retrieval of the manuscript.

- **Minor comment 2**

The terminology has been updated throughout the manuscript, replacing "Joanna Briggs Institute" with "JBI", in accordance with the organisation's current name.

- **Minor comment 3**

The word "international" has been removed from the *Strengths and limitations* section.

- **Minor comment 4**

A reference to the 2016 Claeys–Leonetti law has now been added.

- **Minor comment 5**

We thank the reviewer for pointing this out. The text has been revised accordingly, and the first name of the cited author has been removed.

- **Minor comment 6**

The manuscript has been revised to include the recommended citation for the Rayyan software.

- **Minor comment 7**

We thank the reviewer for pointing out this omission. Citation numbers have been added wherever Peters et al. is mentioned in the manuscript.

VERSION 2 - REVIEW

**Reviewer**	**1**
**Name**	**Bush, Shirley H.**
**Affiliation**	**University of Ottawa, Department of Medicine**

Date **28-Jul-2026**

COI

---

1. The Authors have responded to my comments with their changes to the protocol.

2. The citation (currently #19) in the Reference list for the use of chlorpromazine in dyspnea is not correct. On searching PubMed, I could not find “subcutaneous chlorpromazine for dyspnea in patients with advanced cancer”.

Here is the PubMed link again from my initial peer review:
https://pubmed.ncbi.nlm.nih.gov/7963786/

The 1st author is McIver B (Not Bruera). The Title and volume/ page numbers are different.

3. As per BMJ instructions (https://authors.bmj.com/writing-and-formatting/formatting-your-paper/) : “References should be cited in numerical order (i.e. 1,2,3) in the text.”

The new references appear to have been added to the end of the old Reference list. Instead, they need to be inserted in order of appearing in the protocol.

E.g. In Rationale, the authors write:

“In adult palliative care, antipsychotics are used in several distinct clinical situations, including the management of delirium, nausea and vomiting, pre-existing or newly emerging psychiatric disorders, and, in selected situations, palliative sedation (2–9).

Although these four indications represent the principal therapeutic indications for antipsychotic use in adult palliative care, other less common therapeutic indications have also been described, such as the use of chlorpromazine for the symptomatic management of refractory dyspnea (19).”

This means that the added citation #19, should be renumbered as #10, as citation #9 was the previous citation.

4. I suspect that the reordering of the text/ other new text may have also affected the citation numbering.

E.g. In Rationale, the authors write:

“Delirium is one of the most frequent neuropsychiatric syndromes encountered in palliative care patients (2). Although non-pharmacological measures remain the cornerstone of management, antipsychotics may be considered when these measures are insufficient, particularly in patients with severe agitation posing a risk to themselves or others, or with distressing perceptual disturbances (4,11).”

But there was no previous citation #10.

The authors need to carefully check all the numbers for citations in the body of protocol and their order in Reference List and correct as needed.

VERSION 2 - AUTHOR RESPONSE

- **Reviewer 1 – Comment 1**

The Authors have responded to my comments with their changes to the protocol.

<u>*Response:*</u>

We thank the Reviewer for this positive assessment.

- **Reviewer 1 – Comment 2**

The citation (currently #19) in the Reference list for the use of chlorpromazine in dyspnea is not correct. On searching PubMed, I could not find "subcutaneous chlorpromazine for dyspnea in patients with advanced cancer". The 1st author is McIver B (Not Bruera). The Title and volume/ page numbers are different.

<u>*Response:*</u>

We thank the Reviewer for identifying this error. The incorrect reference has been replaced with the correct citation:

"McIver B, Walsh D, Nelson K. The use of chlorpromazine for symptom control in dying cancer patients. J Pain Symptom Manage**.** 1994;9(5):341-5. doi:10.1016/0885-3924(94)90193-7."

The corresponding reference has been corrected in the manuscript (page 11, reference 12).

- **Reviewer 1 – Comment 3 and 4**

References should be cited in numerical order (i.e. 1,2,3) in the text. The new references appear to have been added to the end of the old Reference list. Instead, they need to be inserted in order of appearing in the protocol […]. I suspect that the reordering of the text/ other new text may have also affected the citation numbering […] The authors need to carefully check all the numbers for citations in the body of protocol and their order in Reference List and correct as needed.

<u>*Response:*</u>

Thank you for this comment. We carefully reviewed the numbering of all in-text citations and the reference list.

In response to your comment, we performed a complete verification of the citation order throughout the manuscript and confirmed that all references are now numbered according to their first appearance in the text, in accordance with the BMJ reference style. We also carefully checked that each in-text citation corresponds to the correct reference in the reference list and corrected formatting inconsistencies where necessary.
