# Supplementary material for: Use of antipsychotics in adult palliative care: a scoping review protocol
Source: BMJ Open. 2026 Aug 24;16(8):e121834. doi: 10.1136/bmjopen-2026-121834 (PMC13504501; doi:10.1136/bmjopen-2026-121834)
Supplement: Supplementary file 2 [file bmjopen-16-8-s001.docx]

## Appendix 1 – PRISMA-P 2015 Checklist

*This checklist was adapted from PRISMA-P according to the recommendations for scoping review protocols proposed by Peters et al. (2022) (25).*

| **Section/Topic** | | **#** | | **Checklist item** | | **Adaptation to scoping review protocol** | | | | | | **Information reported Yes/No** | | | **Line number(s)** |
| --- | --- | --- | --- | --- | --- | --- | --- | --- | --- | --- | --- | --- | --- | --- | --- |
| **ADMINISTRATIVE INFORMATION** | | | | | | | | | | | | | | | |
| **Title** | | | | | | | | | | | | | | | |
| Identification | | 1a | | Identify the report as a protocol of a systematic review | | Identify the report as a protocol of a scoping review | | | | | | YES | | | *Title* |
| Update | | 1b | | If the protocol is for an update of a previous systematic review, identify as such | | If the protocol is for an update of a previous scoping review, identify as such | | | | | | Non applicable | | | |
| **Registration** | | 2 | | If registered, provide the name of the registry (e.g., PROSPERO) and registration number in the Abstract | | If registered, provide the name of the registry (such as JBI) and registration number | | | | | | Not registered – *“Protocol registration” page 9* | | | |
| **Authors** | | | | | | | | | | | | | | | |
| Contact | | 3a | | Provide name, institutional affiliation, and e-mail address of all protocol authors; provide physical mailing address of corresponding author | |  | | | | | | YES | | | *Page 1* |
| Contributions | | 3b | | Describe contributions of protocol authors and identify the guarantor of the review | |  | | | | | | YES | | | *Page 2* |
| **Amendments** | | 4 | | If the protocol represents an amendment of a previously completed or published protocol, identify as such and list changes; otherwise, state plan for documenting important protocol amendments | |  | | | | | | This is the first version of the protocol. Any important protocol amendments will be described in the final scoping review publication, if applicable - *Page 9 : “Protocol Registration”.* | | | |
| **Support** | | | | | | | | | | | | | | | |
| Sources | | 5a | | Indicate sources of financial or other support for the review | |  | | | | | | YES | | | *Page 10 : “Funding”* |
| Sponsor | | 5b | | Provide name for the review funder and/or sponsor | |  | | | | | | None | | | |
| Role of sponsor/funder | | 5c | | Describe roles of funder(s), sponsor(s), and/or institution(s), if any, in developing the protocol | |  | | | | | | Not applicable | | | |
| **INTRODUCTION** | | | | | | | | | | | | | | | |
| **Rationale** | 6 | | | Describe the rationale for the review in the context of what is already known | |  | | | | | YES | | | *Page 2 : “Introduction – Rationale”* | |
| **Objectives** | 7 | | | Provide an explicit statement of the question(s) the review will address with reference to participants, interventions, comparators, and outcomes (PICO) | | Provide an explicit statement of the question(s) the review with reference to the inclusion/exclusion criteria. Scoping reviews may not use PICO and instead may use JBI’s Population, Concept and Contexte (PCC) or another approach to reporting eligibility criteria. | | | | | YES | | | *Page 3 : “Introduction – Objectives”* | |
| **METHODS** | | | | | | | | | | | | | | | |
| **Eligibility criteria** | 8 | | | Specify the study characteristics (e.g., PICO, study design, setting, time frame) and report characteristics (e.g., years considered, language, publication status) to be used as criteria for eligibility for the review | | Scoping reviews may not use PICO and instead may use JBI’s Population, Concept and Contexte (PCC) or another approach to reporting eligibility criteria. | | | | YES | | | *Page 4 : “Inclusion criteria”* | | |
| **Information sources** | 9 | | | Describe all intended information sources (e.g., electronic databases, contact with study authors, trial registers, or other grey literature sources) with planned dates of coverage | |  |  |  |  | YES | | | *Page 6 : “Type of documents” and “Source of documents”* | | |
| **Search strategy** | 10 | | | Present draft of search strategy to be used for at least one electronic database, including planned limits, such that it could be repeated | |  |  |  |  | YES | | | *Page 10 : “search strategy” and Appendix 2.* | | |
| **STUDY RECORDS** | | | | | | | | | | | | | | | |
| Data management | 11a | | Describe the mechanism(s) that will be used to manage records and data throughout the review | | |  | | | YES | | | | *“Methods” – page 7 and 8* | | |
| Selection process | 11b | | State the process that will be used for selecting studies (e.g., two independent reviewers) through each phase of the review (i.e., screening, eligibility, and inclusion in meta-analysis) | | | State the process that will be used for selecting studies (such as two independent reviewers through each phase of the review (that is screening, eligibility and inclusion) | | | YES | | | | *“Methods” – page 7 and 8* | | |
| Data collection process | 11c | | Describe planned method of extracting data from reports (e.g., piloting forms, done independently, in duplicate), any processes for obtaining and confirming data from investigators | | |  | | | YES | | | | *“Methods” – page 7 and 8* | | |
| **Data items** | 12 | | List and define all variables for which data will be sought (e.g., PICO items, funding sources), any pre-planned data assumptions and simplifications | | | Scoping reviews may not use PICO and instead may use JBI’s Population, Concept and Contexte (PCC) or another approach to reporting eligibility criteria. | | | YES | | | | *“Data extraction” – page 8* | | |
| **Outcomes and prioritization** | 13 | | List and define all outcomes for which data will be sought, including prioritization of main and additional outcomes, with rationale | | |  | | | YES | | | | *“Data extraction” – page 8* | | |
| **Risk of bias in individual studies** | 14 | | Describe anticipated methods for assessing risk of bias of individual studies, including whether this will be done at the | | | If this occur, describe anticipated methods for assessing risk of bias of individuals studies, including whether this will be done at the outcome or study level, or both; state how this information will be used in data synthesis | | | YES | | | | No formal assessment of risk of bias of individual studies will be conducted, as this scoping review aims to map the available evidence rather than assess methodological quality. *“Strengths and limitations of this study.” – page 9* | | |
| **DATA** | | | | | | | | | | | | | | | |
| **Synthesis** | 15a | | | | Describe criteria under which study data will be quantitatively synthesized | | Describe criteria under which study data will be presented | YES | | | | | As this is a scoping review, no quantitative synthesis will be conducted. Findings will be synthesized using descriptive and narrative approaches, including tabulation of study characteristics, grouping of results into conceptual themes, and mapping of the available evidence – *“Data synthesis” – page 8* | | |
|  | 15b | | | | If data are appropriate for quantitative synthesis, describe planned summary measures, methods of handling data, and methods of combining data from studies, including any planned exploration of consistency (e.g., *I* ^2^, Kendall’s tau) | | Describe the planned approach to how extracted data will be presented (such as figures, tables, evidence gaps maps) |  |  |  |  |  |  |  |  |
|  | 15c | | | | Describe any proposed additional analyses (e.g., sensitivity or subgroup analyses, meta-regression) | | Describe any proposed additional analyses (such as thematic analyses) |  |  |  |  |  |  |  |  |
|  | 15d | | | | If quantitative synthesis is not appropriate, describe the type of summary planned | |  |  |  |  |  |  |  |  |  |
| **Meta-bias(es)** | 16 | | | | Specify any planned assessment of meta-bias(es) (e.g., publication bias across studies, selective reporting within studies | |  | YES | | | | | No assessment of meta-biases (e.g. publication bias or selective reporting) will be undertaken, as this scoping review aims to map the available evidence rather than evaluate methodological quality. *“Strengths and limitations of this study” – page 9* | | |
| **Confidence in cumulative evidence** | 17 | | | | Describe how the strength of the body of evidence will be assessed (e.g., GRADE) | | If this is to occur, the method should be described | YES | | | | | The certainty of the body of evidence will not be assessed, as this scoping review aims to map the available evidence rather than evaluate the certainty of the evidence. *“Strengths and limitations of this study” – page 9* | | |
