# Supplementary material for: Use of antipsychotics in adult palliative care: a scoping review protocol
Source: BMJ Open. 2026 Aug 24;16(8):e121834. doi: 10.1136/bmjopen-2026-121834 (PMC13504501; doi:10.1136/bmjopen-2026-121834)
Supplement: Supplementary file 3 [file bmjopen-16-8-s002.docx]

## Appendix 2 – Search Strategy for PubMed.

### 09/03/2026.

| #1 | "antipsychotic agent"[MeSH Terms] | 62,880 |
| --- | --- | --- |
| #2 | "dopamine antagonist"[MeSH Terms] | 12,414 |
| #3 | "tranquilizing agents"[MeSH Terms] | 96,744 |
| #4 | **(("Antipsychotic*"[Title/Abstract]) OR ("Neuroleptic*"[Title/Abstract]) OR ("Major tranquiliz*"[Title/Abstract]) OR ("dopamine antagonist*"[Title/Abstract]))** | 73,307 |
| #5 | #1 OR #2 OR #3 OR #4 | 139,233 |
| #6 | "palliative care" [MeSH Terms] | 69,112 |
| #7 | "palliative Medicine"[MeSH Terms] | 652 |
| #8 | "Hospice and Palliative Care Nursing"[MeSH Terms] | 2,657 |
| #9 | **(("palliative car*"[Title/Abstract]) OR ("terminal car*"[Title/Abstract]) OR ("end of life care*"[Title/Abstract]) OR ("Palliative medicine"[Title/Abstract]) OR ("palliative treatment*"[Title/Abstract]) OR ("terminal ill*"[Title/Abstract]) OR ("end of life ill*"[Title/Abstract]) OR ("hospice car*"[Title/Abstract]) OR ("hospice*"[Title/Abstract]) OR ("supportive car*"[Title/Abstract]) OR ("comfort car*"[Title/Abstract]) OR ("End of life"[Title/Abstract]) OR ("palliative stag*"[Title/Abstract]) OR ("terminally ill*"[Title/Abstract]))** | 127,236 |
| #10 | **#6 OR #7 OR #8 #9** | 158,029 |
| #11 | **#5 AND #10** | 785 |
